# Gene drive strategies of pest control in agricultural systems: Challenges and opportunities

**DOI:** 10.1111/eva.13285

**Published:** 2021-08-04

**Authors:** Mathieu Legros, John M. Marshall, Sarina Macfadyen, Keith R. Hayes, Andy Sheppard, Luke G. Barrett

**Affiliations:** ^1^ CSIRO Agriculture and Food Canberra ACT Australia; ^2^ CSIRO Synthetic Biology Future Science Platform Canberra ACT Australia; ^3^ Divisions of Biostatistics and Epidemiology – School of Public Health University of California Berkeley CA USA; ^4^ CSIRO Data 61 Hobart Tas. Australia; ^5^ CSIRO Health and Biosecurity Canberra ACT Australia

**Keywords:** agriculture, gene drive, genetic control, pest control, resistance, risk analysis, underdominance

## Abstract

Recent advances in gene‐editing technologies have opened new avenues for genetic pest control strategies, in particular around the use of gene drives to suppress or modify pest populations. Significant uncertainty, however, surrounds the applicability of these strategies to novel target species, their efficacy in natural populations and their eventual safety and acceptability as control methods. In this article, we identify issues associated with the potential use of gene drives in agricultural systems, to control pests and diseases that impose a significant cost to agriculture around the world. We first review the need for innovative approaches and provide an overview of the most relevant biological and ecological traits of agricultural pests that could impact the outcome of gene drive approaches. We then describe the specific challenges associated with using gene drives in agricultural systems, as well as the opportunities that these environments may offer, focusing in particular on the advantages of high‐threshold gene drives. Overall, we aim to provide a comprehensive view of the potential opportunities and the remaining uncertainties around the use of gene drives in agricultural systems.

## 1 INTRODUCTION

Pest species (i.e. weeds, pathogens, rodents and invertebrate pests) in agricultural systems are responsible for significant economic losses worldwide, estimated to be $540 billion per year if unchecked (Willis, 2017). Pests represent a threat to agricultural productivity and often food safety. Control strategies in modern agriculture are heavily reliant on chemical pesticides, but this approach is facing increasing challenges. The effectiveness of many pesticides is being continually eroded by the evolution of resistance, and there is evidence of environmental or human harm leading to societal opposition in many parts of the world and an increasing list of banned pesticides (Nag & Gite, 2020). Furthermore, new pests are constantly emerging, while existing pests are establishing themselves in new agro‐ecosystems around the world. There is a need for scientific advances to offer novel, sustainable and safer pest control approaches relative to pesticides.

Genetic pest control strategies have been considered for decades as an alternative to chemical approaches and a potential addition to the pest control toolbox (Curtis, 1968; Gould, 2008). In the broadest sense, genetic control strategies use genetically manipulated strains of a pest organism to achieve one of two main objectives: population suppression, aiming at lowering the densities of natural target populations or eliminating them entirely, or population replacement, aiming at replacing natural pests with less damaging individuals. A subset of these approaches, referred to as gene drives, make use of the super‐Mendelian inheritance patterns of selfish genetic elements to drive genetic cargo (i.e. any genetic material of interest, such as a gene conferring resistance to a disease or a gene imposing conditional lethality) into natural populations. These elements are also themselves often referred to as ‘gene drives’, and in this article, we make sure to systematically distinguish between a ‘gene drive element’ (the genetic material with selfish inheritance properties) and a ‘gene drive strategy’ (the pest control approach that aims to suppress or replace populations).

Gene drive elements can be derived from naturally existing selfish genetic elements or engineered from entirely synthetic designs. Gene drive strategies can subsequently be built around a number of gene drive elements (Raban et al., 2020). Importantly, different elements may have inherently different spatial and temporal dynamics. Such differences can significantly influence the design of a gene drive strategy. In this paper, we follow the terminology put forward by Alphey et al. (2020) and distinguish the following types of gene drive elements:

- *Nonlocalized drives* are gene drive elements characterized by a low threshold to their capacity to drive (meaning they can increase in frequency in a population from low initial frequencies) and no inherent limitation to their ability to spread. Examples of nonlocalized drives include natural elements like *Medea*, a selfish genetic element initially discovered in flour beetle (*Tribolium* spp.) populations (Wade & Beeman, 1994; Ward et al., 2011). Other notable nonlocalized drives include a family of synthetic gene drive elements based around the use of site‐directed endonucleases (SDN gene drives) to achieve gene drive (Burt, 2003; Deredec et al., 2008; Esvelt et al., 2014; Noble et al., 2018). This family of gene drives has received considerable attention in recent years, primarily due to the advent of CRISPR molecular capabilities that provide a lot of design and engineering flexibility. These molecular tools can be used to design a nuclease targeting a precise site based on a specifically designed guide RNA (gRNA). Using the cell's homology‐directed DNA repair mechanisms, this synthetic element can be copied on the homologous chromosome and therefore increase in frequency (Esvelt et al., 2014).
- *Localized*, *high*‐*threshold drives* (hereafter referred to simply as ‘high‐threshold drives’) require a high frequency threshold to be able to spread in a population. Because of this, in principle they are restrained in their ability to spread spatially, since an invasion into a neighbouring, nontarget population is likely to start at a low frequency and therefore remain under the high threshold. Examples of high‐threshold drives include natural systems like translocations (Curtis, 1968), as well as synthetic systems such as engineered translocations (Buchman et al., 2018) or engineered underdominance (Davis et al., 2001). It should also be noted that, since the frequency threshold of a gene drive element increases with its associated fitness costs, gene drive elements described above as nonlocalized may be functionally high‐threshold drives if they are associated with sufficiently high fitness costs (Backus & Delborne, 2019)
- *Self*‐*limiting drives* refer to gene drive strategies that are designed to be able to drive only for a limited period of time. Their spread is therefore expected to be temporally and spatially limited. These strategies are typically based on synthetic designs involving two or more independent elements, like Killer Rescue (Gould, 2008) or daisy‐chain (Noble et al., 2019) approaches.

It should also be noted that the gene drive element can be introduced into the target species in various forms. In most cases, the element is directly inserted into the target genome. Another approach, however, involves the use of *Wolbachia* endosymbionts in insects. Because of their inheritance properties, *Wolbachia* can be used for population replacement or population suppression, both as a nonlocalized drive and as a high‐threshold drive (Ross et al., 2019). Finally, there are also strategies of genetic pest management that do not rely on gene drive elements, such as sterile insect technique (SIT) approaches and related strategies like the release of insects carrying a dominant lethal (RIDL; Alphey, 2002), hereafter referred to as *nondrive* approaches.

At the time of writing this article, gene drive elements have been implemented in a handful of species, mostly insects, including diseases vectors with *Anopheles* (Gantz et al., 2015; Hammond et al., 2016; Kyrou et al., 2018; Windbichler et al., 2011) and *Aedes* mosquitoes (Li et al., 2020), agricultural pests with *Drosophila suzukii* (Buchman et al., 2018), and model species with *Drosophila melanogaster* (Chen et al., 2007; Gantz & Bier, 2015; Reeves et al., 2014). Accordingly, applications of gene drive to insect pests, and particularly to mosquito disease vectors, have received most of the theoretical attention (Eckhoff et al., 2017; Legros et al., 2013; Marshall, 2009; North et al., 2013; Robert et al., 2012).

The universality of the CRISPR‐Cas9 gene‐editing system, which in principle can be used as a molecular tool in a very broad range of species, has raised interest in genetic control strategies broadly. As a consequence, and in combination with the conceptually simple CRISPR‐based SDN gene drive system described above, a very large array of undesirable species, some of which may not have been considered amenable to genetic engineering approaches, suddenly emerged as potential candidates for genetic control strategies. Consequently, many questions have been raised regarding the feasibility of gene drive strategies in novel target species, the ideal gene drive elements to be used for each purpose and the impact of biological, ecological and environmental traits on the outcome of a gene drive strategy.

Because of the considerable negative impact of weeds, invertebrate pests and diseases of agriculture, there has been increasingly strong interest in the application of genetic strategies to the control of these organisms. To date, however, there have been very few cases of demonstrable gene drive engineered in agriculturally relevant species. A toxin‐antidote‐based gene drive known as *Medea* has been engineered in *D. suzukii* (Buchman, Marshall, et al., 2018), a significant pest of several species of soft‐skinned fruits. There are ongoing efforts to establish functioning gene drives in rodents (Grunwald et al., 2019), which could be of interest to the grain industry, although there are significant challenges to this task (Pfitzner et al., 2020). Finally, a naturally occurring gene drive has recently been successfully transferred into the globally important cereal pathogen *Fusarium gramineum* (Gardiner et al., 2020).

Given the huge diversity of pest species that impact agricultural production, there is a wide range of obstacles to the use of genetic control methods against agricultural pests. As an example, Barrett et al. (2019) provided an overview of the main challenges faced by gene drive development in weeds, and a similar range of technical and regulatory hurdles can be expected for other taxa like fungal pathogens and nondipteran insects. There has been relatively little consideration for the practical aspects of the application of genetic control strategies in agricultural systems, for the potential challenges that come with these new technologies in new environments or for the opportunities that could present themselves. The most useful exercise at this early stage in the process is therefore to determine which species may have the greatest potential for the successful development of these new tools, which drives are best suited to the goals of agricultural pest control and which areas of uncertainty remain.

The objective of this paper is to summarize the opportunities and challenges associated with novel approaches for genetic control of agricultural pests. We offer a broad framework to assess trade‐offs across biologically diverse pest species and highlight some of the specific attributes of species that need to be understood prior to the development of genetic control programmes. We review how managed systems such as agricultural landscapes may facilitate or impede the implementation of genetic control programmes around gene drives. Finally, we discuss the strategies for gene drive confinement and risk mitigation in agricultural environments and examine the factors that need to be considered to ensure durable and sustainable solutions that may open a new era of pest control in agricultural systems.

## 2 AGRICULTURAL PESTS AND AGROSYSTEMS: THE NEED FOR INNOVATIVE GENETIC CONTROL

### 2.1 Current failures and potential targets in agricultural pest control

Accurate estimates of agricultural losses caused by pest species are difficult to obtain because the damage caused by these organisms depends on environmental conditions, the crop being cultivated, socio‐economic factors and the level of technology used. However, it is generally true that weeds, pathogens and insect pests are major competitors with humans for resources generated by agriculture (Pimentel, 2011) and that they benefit from many conditions associated with modern agriculture, particularly the use of extensive monocultures and the intensive use of fertilizers (Oerke & Dehne, 2004). For example, among the world's most important food crops—rice, wheat, maize, potatoes and soybeans—losses to pathogens are estimated to average 13%–22% annually (Oerke, 2006; Savary et al., 2019). Economic costs associated with insect pests are estimated to be at a similar level overall (Oerke, 2006). Furthermore, the economic costs associated with pests extend beyond the direct loss of yield, including reductions in quality, as well as the costs of management actions (e.g. pesticide application) to minimize losses.

Current control options for pests are increasingly limited by the emergence of populations that have developed resistance to chemical control measures (Gould et al., 2018; see Box 1 for an example). For example, following ~70 years of herbicide use, there are more than 250 cases of evolved herbicide‐resistant weed species encompassing 23 of the 26 known herbicide modes of action (Heap, 2020). For insect pests and pathogens, in addition to chemical options, the breeding of crops for pest resistance and tolerance is an important control technology. However, both modes of control are threatened by evolutionary adaptation in pest populations (Burdon et al., 2016; Fisher et al., 2018; Sparks & Nauen, 2015). These problems are compounded by the fact that the rate at which new chemistries and resistance genes are brought to market has slowed to a trickle and lags behind the rate at which evolution in pest species occurs.

Such evolution, coupled with a paucity of new genetic and chemical options, poses significant risks to future food security. Furthermore, pesticides face increasing scrutiny from the public and regulators due to human health and environmental risks, with the potential for restriction on use or outright ban of certain products. New, sustainable tools for pest control must offer safer and lower risk alternatives to existing pesticides. Furthermore, new approaches must learn from failures during the development of GM approaches in the 1990s. Tools providing the capacity to directly manipulate the genetics of pest populations are one potential solution. While it seems unlikely that any new tools will be free from problems associated with pest evolution, genetic control strategies do offer some potential advantages. In particular, genetic control is by definition highly specific to a given pest species or biotype and is likely to have relatively low levels of nontarget toxicity (toxic impacts on nontarget organisms) compared to chemical approaches.

### 2.2 Innovative control in agriculturally relevant pests: history and outlook

The development and dissemination of modern pest control strategies, primarily chemical pesticides and genetic resistance bred into crops, transformed agriculture in the 20^th^ century. Chemical pesticides and host plant resistance are currently favoured due to ease of use and economic efficiency in most systems, although pesticides can be associated with complex and sometimes hidden costs (Bourguet & Guillemaud, 2016). However, there are many potential alternatives. Biological control, (the use of naturally occurring predators, parasitoids and diseases as agents to attack pests) for example, has a long history of application in agriculture. Classical biological control has had some notable successes in widespread sustained suppression of major pests, such as the citrus red scale *Aonidiella aurantii* in California (Reeve & Murdoch, 1985) and prickly pear *Opuntia* spp. control programmes in Queensland, Australia (White, 1980). Classical biological control offers a low‐input means of sustained pest management, but can be variable in efficacy and applicability. Augmentative biological control using generalist natural enemies is now standard practice as part of integrated invertebrate pest management for glasshouse crops (Gullino et al., 2020), but is not as effective in broadacre agriculture, where pesticides predominate (Pretty, 2005).

Biocontrol approaches based on genetic manipulation of pest populations (Gould, 2008) started with sterile insect technique (SIT), a species‐specific method for insect population control that relies on genetic disruption using irradiation to create and mass rear sterile insects for release into target populations (Knipling, 1959). Released insects compete for mates with wild males; a wild female mating with a released sterile male has no or fewer progeny, leading to population suppression (Dyck et al., 2005). Applied across a landscape and in sufficient numbers, SIT can provide an effective means of area‐wide pest control and is successfully being applied worldwide to control Mediterranean Fruit Fly, the screwworm fly and the tsetse fly (Klassen & Curtis, 2005).

Advances in molecular biology have resulted in the development of SIT‐type strategies with increasingly sophisticated tools for conferring (and perpetuating) sterility, as well as engineering sex‐specific lethality or facilitating sex sorting when the release of a single sex is required (Alphey, 2002). For example, in a female‐specific RIDL approach (fsRIDL), genetic engineering is used to engineer male insects carrying female‐specific lethality genes (Fu et al., 2010; Schetelig et al., 2021). Like SIT, males are mass‐reared and released. Unlike SIT, the transgene is only lethal in female offspring and is passed on to viable male offspring (although does not drive) who can in turn cause female‐specific lethality in subsequent generations.

Genetic engineering has led to significant advances in pest control. The engineering of resistance into plants has proven effective for some crops, like *Bt* cotton (Downes et al., 2017; Wilson et al., 2018), although there has been many instances of resistance evolution against *Bt* crops (Tabashnik & Carrière, 2019). Advances in genetic engineering and the understanding of gene regulation have led to substantial research into pest control options which directly target pest genetics. Gene silencing strategies based on RNA interference (Kim & Rossi, 2008) use hairpin RNAs or small RNAs to interfere with gene expression at the transcriptional level. This mechanism is conserved across eukaryotes, so in principle can be designed to target any pest species of interest. While initially developed as a passive resistance mechanism, whereby hosts are engineered to express RNAi constructs, more recent studies demonstrate the potential for active population control via topical application of RNA molecules directly onto pests, also known as spray‐induced gene silencing (SIGS; Wang & Jin, 2017). This approach, while promising, has significant challenges to overcome regarding the delivery and stability of the molecule following application, uptake by the hosts and pests, and systemic movement of the siRNA within the host, but promising advances have been made in targeting plant pathogens (Mitter et al., 2017).

BOX 1 **Strategies and challenges for aphid pest control.**

Aphid pests are some of the most challenging invertebrate pest species to manage. They can cause yield loss through direct feeding on crops, and their population densities can increase rapidly through asexual reproduction during suitable environmental conditions. Importantly, they can vector a range of damaging plant diseases. Aphid‐resistant crop varieties have yet to be developed, so insecticide applications and natural enemies (predators and parasitic wasps) remain widely used control methods. In Australian canola and pulse crops, *Myzus persicae* (the green peach aphid) has developed resistance to carbamates, pyrethroid and neonicotinoid insecticides (Edwards et al., 2008). Resistance to organophosphates has been observed in populations, and there is some evidence of resistance to neonicotinoids (de Little et al., 2017; de Little & Umina, 2017; GRDC, 2015; Umina et al., 2014). The economic costs of aphids to crop productivity and farm incomes are substantial (Valenzuela & Hoffmann, 2015), and the development of resistance is making insecticide control options more limited. There is some evidence that climate change may also speed up the rate of evolution of resistance for some invertebrate pests, for example red‐legged earth mites (Maino et al., 2018). Furthermore, the use of insecticides has many nontarget impacts (Roubos et al., 2014) and can make managing a suite of pest species present in the crop challenging due to the removal of natural enemies and potential secondary pest outbreaks (Hill et al., 2017; Wilson et al., 1999).

## 3 THE IMPORTANCE OF ECOLOGY AND LIFE HISTORY FOR THE OUTCOME OF GENE DRIVE CONTROL STRATEGIES

Given the diversity of significant target pests that would benefit from innovative control solutions in agricultural systems, the versatility of CRISPR‐Cas9 gene‐editing technology is a key feature. However, the diversity of potential target species generates very high levels of uncertainty about the impact of species‐specific traits on the fate of a hypothetical gene drive in their natural environment. While the general importance of some traits like dispersal and fitness costs on various gene drives is well understood (Backus & Delborne, 2019; Legros et al., 2013; Noble et al., 2018), the range of potentially important species‐specific traits affecting a gene drive for all potential target species is much wider and still largely unknown. Understanding how biological and ecological traits impact the outcome of a gene drive (i.e. the success or failure of a gene drive strategy) could facilitate the screening of pest species to identify their potential as targets for genetic management via gene drive (or their unsuitability for such methods). In this section, we do not look to provide an exhaustive list of features that may impact the fate of a gene drive, but aim instead to place a spotlight on important features of agriculturally relevant pests across taxa, including insects, weeds and fungal pathogens, and emphasize how they might impact the outcome of a gene drive, by either facilitating the goal of genetic pest control or posing a significant challenge to its feasibility.

### 3.1 Dispersal and gene flow

Dispersal is defined as the movement and subsequent reproduction of individuals from one area to another, sometimes involving long distances (e.g. migratory flights), and has a strong influence on the population dynamics of species and genetic structure of populations (Stinner et al., 1983). Dispersal is one of the most influential traits for genetic control strategies, as it affects (a) the ability of transgenes to permeate the target population and control area, (b) the risk of transgenes spreading to nontarget populations (and possibly to nontarget sexually compatible species) and (c) the resources required to adequately monitor outcomes following release. Regardless of the species of interest, dispersal is often mathematically described by a long‐tailed, or leptokurtic, distribution. This reflects the assumption that dispersal is most often relatively local, while dispersal over larger spatial scales occurs more infrequently (e.g. via human, wind or other assisted pathways). Local gene flow is crucial for the success of a genetic control strategy, while the extent of large‐scale movements and dispersal events is important to consider for confinement and biosafety.

Local gene flow directly impacts the ability of a gene of interest (like a gene drive) to permeate and spread through a target area. Models of gene drive strategies in mosquito populations show that local dispersal is one of the most important factors in predicting the outcome of a gene drive control programme (Xu et al., 2010). Local gene flow is also the primary factor in predicting the ability of a gene drive to spread among neighbouring populations (whether that is an intended or unintended consequence of a release; Noble et al., 2018). Consequently, a precise knowledge of the dispersal abilities, patterns and potential barriers to dispersal for a target pest will be required to predict the outcome of any gene drive control strategy. For instance, recent studies of the Asian citrus psyllid (*Diaphorina citri*), the vector of citrus huanglongbing (citrus greening disease), point to movement of psyllids between citrus fields several kilometres apart, especially during summer months, with psyllids traversing barriers such as roads and fallow fields (Lewis‐Rosenblum et al., 2015). This behaviour will affect both the ability of a gene drive to spread through a target area and the probability that a gene drive (particularly a nonlocalized drive) might spread to unintended areas.

For many agricultural pests, local dispersal patterns vary across seasons depending on environmental conditions and resource availability. Studies of *D. suzukii*, a major pest of fruit crops in Europe and the United States, demonstrate movement dependent on seasonal fruit availability. In California's Central Valley, for instance, *D*. *suzukii* populations peak in cherry orchards leading up to their harvest in June, after which they disperse to other citrus crops and noncrop habitats (Wang et al., 2016). Similar movement between crop and noncrop habitats was observed in cherry orchards in the Verona Region of Italy (Tonina et al., 2018). This behaviour will affect the optimal timing and spatial targeting of a genetic control programme against this particular pest.

Long distance dispersal (LDD) is a common trait among many pest species. For example, LDD can spread plant diseases across continents and consistently re‐establish populations following local extinctions (Zeng & Luo, 2006). The lepidopteran insect pest, fall armyworm (*Spodoptera frugiperda*), has the capacity to migrate more than 500 km per generation (Westbrook et al., 2016), driving the rapid invasion of this species in many new ranges around the world. Even for species lacking specific adaptations, uncommon weather events (Stern, 1979), escalating globalization and trade provide means for agricultural pest species to disperse long distances. LDD will likely present challenges for genetic management using gene drive. The strongly stochastic nature of LDD means that individual events will often defy prediction and are likely to cross geographic borders, creating issues relating to monitoring, confinement, regulation and biosafety. LDD also has potential to influence spatial genetic structure. In particular, the genotypes that disperse to new territories may be atypical and not representative of the source population, creating issues for strategies that are targeted to specific genotypes.

Accurate forecasting of dispersal and drive in many pest species may require the inclusion of additional nuance relating to habitat distribution, which is often modelled as a continuous landscape. For example, weed dispersal is often modelled based on a leptokurtic kernel (Bullock et al., 2017), with frequent short‐range dispersal events (on the order of 2 m) and infrequent long‐range events (detected up to 100 m from the sources in one study; Skarpaas & Shea, 2007). When applied to a continuous landscape, this manifests as an invasion progressing with constant speed, an unlikely scenario outside of a model. For underdominant genetic control systems (characterized by high‐threshold frequency‐dependent drive ability, see Section 4), this may lead to unrealized predictions of progressive spatial spread in the form of an advancing hybrid zone (Barton & Turelli, 2011; Champer et al., 2020). More precise information on pest habitat distribution, dispersal rates and potential barriers to gene flow will help to make more accurate predictions regarding the expected spread of gene drives, the outcome of genetic control programmes and the associated confinement and biosafety procedures.

### 3.2 Population dynamics, demography and age and stage structure

Population dynamics and the age and stage structure of a target species are often linked, and the resulting variations in temporal and spatial patterns of population densities and age distribution can influence the timing and nature of selective forces in a target population. However, these variations are often highly specific to individual pest species and life histories. Genetic control strategies are highly sensitive to the relative fitness profiles of released, drive‐carrying individuals and resident, wild‐type individuals (Backus & Delborne, 2019), which typically define the critical release ratios needed for a successful implementation (whether the goal is population replacement or suppression). The specificities of a target's population dynamics and demography are therefore highly relevant to any genetic control programme, and we illustrate in this section how they are likely to affect (a) the optimal timing of the release(s) of gene drive‐carrying individuals, (b) the numbers of individuals to be released and their spatial distribution and (c) the outcome of releases across different ages or stages of the target population.

The population dynamics of most agricultural pest species are defined by resource availability (often crops) and environmental conditions. Given the seasonal nature of many crops, pest population sizes often fluctuate tremendously in consequence. For example, for *D*. *suzukii* and several other fruit crop pests, population dynamics are strongly linked to temperature and fruit quantity and quality during the off‐season. Off‐season resources drive the population dynamics by influencing mortality and development rates of all life stages (Gutierrez et al., 2016; Langille et al., 2016). Temperature also influences female fecundity, and over the winter months, *D*. *suzukii* undergoes a reproductive diapause (Emiljanowicz et al., 2014; Ryan et al., 2016). Consequently, the population densities of a *D*. *suzukii* population are expected to not only fluctuate between seasons, but to do so in a different fashion and with a different temporal profile across years depending on environmental factors affecting off‐season resources. Because a potential population suppression programme against this pest will work best if it targets the population at its lowest density, it is necessary to understand the dynamics of all stages of a population on any given year to be able to optimize the reduction in crop damage from a given release implementation (numbers released and spatial pattern).

Similarly for population replacement approaches, the dynamics of the target species may impact the fate of a released replacement strain. The Asian citrus psyllid, for instance, is a potential target for such strategies, by introducing refractory genes that prevent transmission of citrus huanglongbing (Baltzegar et al., 2018; Chiyaka et al., 2012). Seasonal fluctuations in psyllid population size are thought to result from the growth of new flushes on citrus trees because the adults lay their eggs here and larvae develop exclusively on the younger parts of citrus trees (Hall et al., 2013). This leads to large infestations of psyllid populations occurring in late spring through mid‐summer. For a population replacement control programme aiming to introduce huanglongbing‐refractory genes into a psyllid population, understanding the precise timing of such population fluctuations will be required. This is because the numbers of released individuals required to achieve an optimal ratio of gene drive to wild‐type individuals will be contingent on the amplitude of such fluctuations and the availability of mating adults. The ability to predict the population dynamics of this pest (or any other) will be crucial to optimize the implementation of a genetic control strategy.

Genetic bottlenecks may also constitute a critical issue for the many pest species undergoing seasonal ‘boom and bust’ demographic dynamics. The strongly stochastic nature of survival during seasonal bottlenecks reduces the effective size of populations and creates potential for strong genetic drift and founder effects between generations and among populations (Barrett et al., 2008). As a consequence, the probability of certain genotypes of interest (e.g. drive carriers or target sequences for SDN drives) persisting among seasons may be affected.

Understanding population dynamics across all stages and their role in regulating population size is essential to predict and evaluate the impact of a population suppression technology. This concept is well illustrated by situations where density‐dependent mortality or competition in specific life stages affects the ability to suppress populations of the later stages (Legros et al., 2009; Phuc et al., 2007). For example, for mosquito vectors of human diseases, density‐dependent competition occurs at the larval stages. Hence as the population is suppressed, density‐dependent mortality decreases; therefore, larval survival increases and suppression of adult stages becomes more difficult (Deredec et al., 2011). In plants, the existence of stage structure in the form of seed banks has a strong influence on control approaches for the above‐ground population. Recent modelling has shown that, as seed banks increase in depth (i.e. increasing average time to germination), there are negative impacts on the use of a gene drive for population suppression (Barrett et al., 2019). First, the spread of the drive allele in the population is delayed, as a result of immigration from the seed bank into the above‐ground population, whereby the seed bank essentially acts as a reservoir for wild‐type alleles. Second, the resulting fitness impact and population suppression effect is similarly delayed. On the other hand, for a temporally limited gene drive (e.g. over a single season), the seed bank could in turn act as a reservoir for gene drive alleles that could invade wild‐type populations in subsequent seasons. While often subtle, such links between ecology, demography and genetics may make or break a genetic control programme.

### 3.3 Mating systems

Agricultural pests are an extremely heterogeneous group of organisms that encompass the full diversity of reproductive strategies. Furthermore, sexually reproducing species may tolerate very different levels of inbreeding, ranging from primarily self‐fertilized through to highly outcrossing. Indeed, many pest species are highly flexible in this respect. For example, many fungal pathogens can reproduce sexually, asexually and clonally, and sexual reproduction may occur via both self‐fertilization and outcrossing. Similarly, many insect species (e.g. aphids) can reproduce both sexually and parthenogenically, depending on environmental conditions. In plants, the majority of species are hermaphroditic, some are apomictic and levels of inbreeding are often highly variable between species and even populations (Whitehead et al., 2018).

As a general principle, the ability of many gene drives to spread through a target population relies on both sexual reproduction and generation of heterozygotes for transmission. In particular (assuming transmission via sexual reproduction), because the super‐Mendelian inheritance of SDN gene drives is expressed in heterozygotes, the dynamics of such a gene drive construct (in sexually reproducing populations) will be affected by factors that influence standing heterozygosity levels, notably inbreeding patterns. The implications of this type of mating system plasticity for the success and failure of genetic control strategies are not well explored, yet it might impact the ability of a gene drive to spread in two ways. First, the existence of high levels of inbreeding in natural populations of a target species, and the associated reduction in heterozygosity, might impede the ability of a gene drive strategy to simply function and spread, since many gene drive mechanisms act in heterozygotes. Second, the importance of these traits provides an avenue for the evolution of resistance against a gene drive, by potentially selecting for high levels of inbreeding and/or selfing (Bull, 2016; Bull et al., 2019). This is likely to be of particular relevance in the case of hermaphroditic plant and fungal targets, where self‐fertilization can lead to significant levels of inbreeding, and is therefore likely to hinder the ability of gene drive systems to spread in such populations. This could cause a lot of pest species to be ruled out as viable targets for control via gene drive, at least in the first instance.

## 4 OPPORTUNITIES, CHALLENGES AND OPTIONS FOR GENETIC CONTROL IN AGRICULTURAL SYSTEMS

Gene drives are being considered as potential solutions to a wide range of problems, from the control of vectors of human disease to the management of invasive species. While the development of gene drives designed for agricultural purposes would undoubtedly follow in the tracks of some of these more advanced projects, there are properties unique to agricultural systems that would present both specific challenges and opportunities (the focus of this section). Because the dynamics of gene drives are inherently most relevant across populations and at the landscape level, we focus primarily on large‐scale, broadacre farming systems, though gene drives might be relevant for smaller scale production systems (e.g. horticulture).

### 4.1 Opportunities for gene drive implementation in managed systems

Agricultural systems are, by definition, heavily managed. The diversity of these environments notwithstanding, they offer several common advantages for genetic control. First is a logistical advantage: genetic strategies, including gene drive approaches, require manipulation of population densities and genetic frequencies at a large spatial scale, whether it is the release of large numbers of engineered individuals or the associated suppression of resident populations with conventional control methods prior to the release. Managed systems are easier to access, monitor and manipulate (for experimental, management or risk mitigation purposes) at least compared to natural environments that other gene drive targets typically occupy.

**FIGURE 1:**
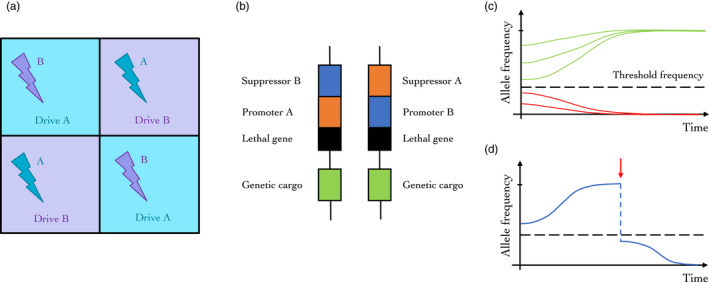
Examples of gene drive approaches in agricultural systems. (a) Example of spatial management of selection pressures in agricultural landscapes. Gene drives can be used to drive traits of interest like sensitivity to a specific biocide (‘Drive A’), while another biocide is used to control pests in the short term (‘
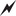
B’). Selection pressures on the drive as well as on the biocide sensitivity can be managed at the landscape levels using spatio‐temporal strategies such as rotation, mosaics (shown here) or mixtures. (b) Example of a high‐threshold gene drive: engineered underdominance (adapted from Davis et al., 2001). Two constructs are engineered and inserted independently into the target genome. When both are present in an engineered organism, both suppressors are active and prevent the expression of the lethal gene. When only one construct is inherited, the lethal gene is expressed and the individual is not viable. (c) Engineered underdominance population genetics: the dynamics of these constructs is characterized by the existence of a threshold frequency (in the case of the specific constructs presented in b, and in the absence of fitness costs, this frequency is 27%; Davis et al., 2001). When the engineered constructs are present in the population at a frequency higher than this threshold, they are driven to fixation (green trajectories). When their frequency is below the threshold, they are driven to extinction (red trajectories). (d) A recall or reversal strategy for high‐threshold gene drives: even after an underdominant gene drive has been driven to fixation, it could be selected against by driving their frequency below the threshold, for example by releasing large amounts of wild‐type individuals (or of another engineered line) in the population (red arrow)

The access to population‐level manipulations opens opportunities to implement sophisticated strategies to promote gene drive spread and confinement, via the control of selective pressure in time and in space. This is the basis for the success of other landscape‐scale programmes, notably the deployment of GM crops for resistance to insect pests (Brookes & Barfoot, 2015). The durability of these resistance traits is strictly dependent on the ability to understand and manipulate the spatial selective landscape in the pest populations, by creating refuges that maintain a selective advantage for wild‐type individuals (Gould, 2000). Similarly, the durability of fungal pathogen resistance traits in crops is heavily impacted by temporal and spatial patterns of deployment (Rimbaud et al., 2018). Resistance has already been proven to be a significant concern for many types of gene drives (KaramiNejadRanjbar et al., 2018), and spatial patterns of deployment, for single or multiple releases of engineered individuals, will be crucial for the long‐term fate of a gene drive (Dhole et al., 2018; Huang et al., 2011). Potential strategies include directed spatial deployment specifically timed releases, and staggered or mixed releases of several gene drives, in a fashion similar to the current protocols for the deployment of biocides, antibiotics or disease resistance genes (Figure 1a). The level of control afforded by managed systems will therefore likely provide a more favourable environment compared to natural and less easily manipulated populations.

In addition, there will also be the opportunity to manipulate selection pressures in ways that can directly help the spread of the gene drive or help recall a gene drive if necessary. A simple example would be to include, within a specific drive, an allele conferring sensitivity to a specific biocide. Such a sensitizing drive could be allowed to spread through a given population unimpeded by withholding the selective agent (Figure 1a). Gene drive‐carrying individuals can then subsequently be targeted using the corresponding biocide, allowing for a more effective and precise control of the target population without adversely impacting nontarget species (although the complete eradication of the target population will still likely face technical difficulties in the field and might be impossible in practice).

The success of such a strategy likely relies on the absence of significant fitness costs in the absence of the chemical (see (Chae et al., 2020) for an example in *Drosophila*). Costly drives or cargo will likely select for the rapid emergence of resistance or mutations inactivating this cargo. However, if these issues can be overcome, the potential benefits of such a drive would include the following: (i) unintended spread to neighbouring populations could be prevented by using the selective agent; (ii) the elimination of the targeted pest could be triggered by the application of the selective agent, against a population rendered entirely susceptible thanks to the gene drive, and (iii) the same selective agent would represent a targeted mechanism for the recall of the gene drive if desired. Here again, managed agricultural systems represent a simplified environment that will help enable this sort of evolutionary management in time and space. Perhaps this will be most useful in the early stages of gene drive development, when specific constructs reach the point of being tested in large‐scale field trials. The ability to control the selective landscape into which a gene drive is released, and to contain and recall a drive if needed, would undoubtedly constitute required features for this type of trial, although other approaches for recalling a gene drive have been proposed and would be applicable (Rode et al., 2020; Vella et al., 2017).

Finally, a major advantage of gene drive approaches in agricultural systems is to help reduce the reliance on pesticides. In a context where resistance to insecticides, fungicides and herbicides is increasing, and posing major issues for agricultural pest control around the world, there is a strong incentive for the development of innovative solutions that reduce the reliance on chemicals (Gould et al., 2018). In addition, there is a strong societal desire to reduce the use of chemical biocides, which in turn would likely be economically beneficial to farmers (though the economics of a gene drive control programme are at this stage entirely undefined). Overall, this reduction in pesticide use could promote the acceptability of gene drives in agricultural systems, though of course gene drives are associated with their own set of social and regulatory challenges (see Section 4.3). Given that a number of agriculturally relevant pests are also invasive species (Paini et al., 2016), gene drives that target invasive pests may have ecological as well agricultural benefits and may therefore be more acceptable (Jones et al., 2019).

### 4.2 The case for high‐threshold drive systems

A high‐threshold gene drive element can be simply defined as any gene drive that can only spread when present in a population at a frequency exceeding a given, high level (Alphey et al., 2020). However, categorically defining which drive systems may act as high‐threshold drives can be difficult, because most drives would be subject to high frequency thresholds when they confer high fitness costs. While the argument could conceptually be extended to any drive with an introduction threshold resulting from such costs, the uncertainty and variability associated with such costs make them extremely challenging to evaluate before release, and the corresponding frequency thresholds in natural populations are both spatially and temporally variable and dependent on environmental factors (Backus & Delborne, 2019). To clarify, we will focus in this paragraph on gene drives that are threshold‐dependent even in the absence of fitness costs, such as engineered underdominance (Figure 1b,c), which comes in many possible forms (Davis et al., 2001; Magori, 2005), or translocations, natural or engineered (Buchman, Ivy, et al., 2018; Curtis, 1968; Gould & Schliekelman, 2004).

High‐threshold drives confer several advantages from a regulatory perspective. For populations with sufficiently large threshold frequencies and limited rates of exchange with nontarget populations, dispersal is unlikely to result in uninhibited spread of the drive. This is because the drive system will be present at a frequency below the threshold in nontarget populations. At such frequencies, the gene drive system and associated transgenes are actively eliminated (Figure 1c) and risks associated with unintended spread are significantly reduced. Similarly, for a gene drive that does not significantly impact population size (e.g. for population replacement approaches), the target population will be resilient to the invasion of wild‐type individuals from neighbouring populations if the gene drive is established at high frequencies. For suppression strategies, on the other hand, high‐threshold gene drives might allow for a locally targeted population suppression but will then leave the area vulnerable to re‐invasion by wild‐type individuals (Greenbaum et al., 2021), unless gene drive individuals are regularly released.

The motivation for high‐threshold drives is enhanced by the managed nature of agricultural systems. First, it is likely much easier to manipulate genotypic frequencies in a specific target population in a managed vs. a nonmanaged system. This includes the ability to lower resident frequencies prior to the release of the drive (e.g. pesticide application), increased logistical capabilities to release high numbers of the drive individuals and, importantly, an improved ability to achieve high levels of spatial coverage, a crucial factor for the establishment of a high‐threshold drive (Legros et al., 2013).

Second, many pest species are defined as such only within the context of agriculture. In other contexts (e.g. natural ecosystems within endemic range), they may not be considered undesirable at all. This makes the ability to limit the spread of a gene drive in time and space critical. Several high‐threshold gene drives are particularly suitable in that regard since, given a high frequency required for drive, any unintended release or movement out of a target area would likely lead to a sub‐threshold presence in nontarget populations.

Third, for many potential target species, the engineering of self‐sustaining drives based on the use of site‐directed nucleases might prove very challenging. In plants for example, there are several significant hurdles to the potential development of such drives, including a higher prevalence of repair pathways of double‐stranded DNA breaks that do not rely on homology and therefore hinder the feasibility of SDN drives as tools for weed control (Barrett et al., 2019). Underdominant systems, whether they rely on translocations or on more elaborate artificial constructs like engineered underdominance, are not reliant on these DNA repair pathways. In novel target species where the DNA repair pathways are either unfavourable or unknown, synthetic underdominant drives are therefore likely to be less challenging to design and engineer.

Fourth, high‐threshold gene drives have the benefit that they can be eliminated once a trial is complete, an intervention is no longer needed, or in the event of an unintended consequence or shift in public opinion. Releases of wild‐type individuals can be used to dilute the gene drive system to sub‐threshold levels, after which it is actively driven out of the population by the same mechanism that drove it in (Figure 1d). If it is undesirable to release large numbers of pests to dilute the transgene, this could be carried out following a population suppression effort, for instance achieved through insecticides or other conventional control methods. The amount of effort required to lower the population density to the required levels will depend on many factors regarding the target species and its environment, though generally speaking it may be more achievable for an agricultural pest where the environment is more suitable for large‐scale control application for prerelease population suppression.

For all these reasons, we argue that high‐threshold gene drives are well suited to the control of agriculturally relevant pests, and furthermore, that agricultural systems constitute an optimal environment for the testing and implementation of such drives. In other environments and for other objectives, such as disease control or invasive species management, nonlocalized drives like SDN drives may be preferred, but the perspectives of gene drive applications for pest or weed control provide us with a unique opportunity to select gene drive designs that are optimally suited to the biological, ecological, social and regulatory aspects associated with the control objectives.

### 4.3 Prospective biological and ecological challenges for genetic control strategies

The feasibility of engineering synthetic drive systems in novel target species is mostly unknown, as they often require a deep knowledge of the target genome and the identification of specific promoters, acting for example during gametogenesis or during early zygotic development. As noted above, there have been very few cases of demonstrable gene drive engineered in agriculturally relevant species to date, and there are clear challenges associated with developing gene drives in new target species. For instance, when dealing with nonmodel species, the lack of a precise knowledge about genomes, gene expression patterns and suitable promoters will increase the difficulty of designing robust gene drive systems. Uncertainties about population genetic and genomic structure can also substantially impact the efficacy of a gene drive (as described in Section 3). Overall, the idea of applying gene drives to entirely novel phyla presents some formidable challenges. A comprehensive review of these challenges in plants has been published elsewhere (Barrett et al., 2019), as well as a specific focus on the technical aspects of genetic engineering in plants (Kumaran et al., 2020), and the same level of difficulty and uncertainty can be expected when dealing with new taxa, such as fungal pathogens or nondipteran insects.

Gene drives are associated with a number of general challenges, and the roadmap to gene drive development will be long and complex, for agricultural systems as for any other system (Courtier‐Orgogozo et al., 2017; NASEM, 2016). A specific point of contention for agricultural gene drive will likely arise in situations where a species is considered a pest for agriculture, but is not an undesirable species in other environments. With ryegrass (*Lolium* spp.), for instance, the same species can be a significant weed in a given field, and an important pasture crop in a neighbouring farm or even a neighbouring field of the same farm. This will place an even stronger incentive on the design of locally restricted gene drives that can easily be contained or reversed.

Finally, it should be noted that the diversity of species that might be considered as potential targets for genetic control will inevitably be synonymous with an equally considerable diversity of challenges to the development and feasibility of these methods. Gene drive applications are likely to be largely different between animals, plants and fungi and face specific challenges for each of these taxa. In plants, a number of challenges have been described, related for instance to non‐homology‐driven DNA repair pathways (seemingly more frequent in plants), seed banks and their consequences on population genetics, or selfing and other sources of inbreeding in natural populations (Barrett et al., 2019). As another example, the outcome of spore killing gene drives in ascomycete fungal pathogens is dependent on complex mating systems as well as on the precise mechanisms of spore formation after meiosis (Martinossi‐Allibert et al., 2021). Overall, this emphasizes that gene drive approaches face significant hurdles before they can be considered as a suitable option for agricultural pest control, not all gene drive strategies might be suitable for this purpose, and not all target species might be amenable to these strategies.

### 4.4 Risk analyses and confinement strategies

If and when gene drive systems in an agricultural pest are shown to be technically feasible (i.e. proof of a successful population replacement or suppression in strictly contained laboratory experiments), the risks associated with any type of field deployment of this new system will need to be systematically analysed. In our experience, there is little to be gained from conducting a complete risk assessment prior to this point because uncertainty about the transgenic product, its characteristics and the specific context of any proposed release dominate the analysis. These issues need to be carefully circumscribed if the analysis is to be used in a risk‐based decision‐making process. It is important therefore that up to this point risk (through premature escape) is carefully managed through multiple forms of containment (Akbari et al., 2015).

An exhaustive list of the potential risks associated with such a strategy would be beyond the scope of this review, particularly considering that the specific risks are likely to differ widely depending on the target species and between plants, insects and fungal pathogens. Instead, we aim here to provide an overview of the likely process of risk assessment for a gene drive in agricultural settings. We also note that, to echo the discussions in previous parts of this article, we focus on risks associated with biological and ecological aspects of the gene drive strategy and the targets species. Social, societal and economical risks can however be significant when it comes to gene drive approaches, and these risks will need to be an integral part of the risk assessment process (NASEM, 2017) or addressed within a complementary impact assessment (WHO, 2021).

Risk assessments for proposals to deploy gene drive‐modified organisms in agricultural settings will likely follow the same steps advocated for genetically modified organisms more generally, namely (i) problem formulation and hazard analysis; (ii) risk calculation and uncertainty analysis; and (iii) risk characterization, mitigation and monitoring design—ideally within some of phased‐release strategy that is inclusive and participatory (Hayes et al., 2018; Kuzma, 2019; NASEM, 2016; Stirling et al., 2018).

The scope of these risk assessments, the hazards that they address and their specific assessment endpoints, will ultimately be determined by the requirements of national biosafety legislation and will therefore vary on a case‐by‐case basis. The scope, however, should also be informed by two additional sources of information: (a) guidance published by respected organizations, such as the secretariat of the Convention on Biological Diversity (CBD) (CBD, 2016), the European Food Safety Authority (EFSA) (EFSA, 2013, 2020), the World Health Organization (WHO) and Foundation for the National Institutes of Health (FNIH), and the National Academies of Sciences, Engineering, and Medicine (NASEM) (NASEM, 2016); (b) the relevant scientific literature including published outcomes of problem formulation workshops (Benedict et al., 2008; Connolly et al., 2021; David et al., 2013; Hayes et al., 2018; James et al., 2018, 2020; Roberts et al., 2017); and (c) the outcomes of the participatory process and concerns expressed by affected communities, different stakeholder communities and the broader public (Finda et al., 2020; Teem et al., 2019).

Discussions around the potential risks associated with the field release of gene drive‐modified organisms have (almost exclusively) focused on disease vector (mosquito) control, with only limited discussion to date on agricultural applications (Romeis et al., 2020). Nonetheless, many of the key issues are generic, and therefore relevant to the broader context, and can be usefully summarized under five topic headings (Table 1): 1. Spread and persistence of the genetic construct; 2. Impacts on human and animal (pets and livestock) health; 3. Impacts on target populations; 4. Impacts on nontarget populations (including the biodiversity implications of these impacts); and 5. Stability considerations over evolutionary relevant time frames.

**TABLE 1:** Summary of key issues potentially associated with the release of gene drive‐modified organisms

Topic	Potential environmental risk assessment issues
1. Construct spread and persistence	(a) Change in biocide resistance; (b) Change in fitness; (c) Predicted geographic range; (d) Effects following introgression of transgene in sexually compatible species; (e) Effects following horizontal transfer of transgene to other organisms
2. Human and animal health	(a) Change in pathogenicity or vector competence (target and nontarget pathogens); (b) Toxicity and allergenicity; (c) Epidemiological efficacy; (d) Impacts on pets or livestock
3. Target populations	(a) Failure (or partial failure) to achieve target outcomes; (b) Niche replacement; (c) Adverse effects due to altered genetic diversity of a laboratory reared population; (d) Changes in agricultural or land management practices
4. Nontarget populations	(a) Impacts on threatened or endangered species; (b) Adverse changes to food webs (e.g. through competitive or trophic interactions); (c) Impacts on ecosystem services; (d) Effects on other pests and diseases
5. Stability over evolutionary relevant time frames	(a) Construct stability; (b) Population dynamics over evolutionary relevant time frames; (c) Synergistic genetic interactions and consequences of multiple ‘stacked’ transgenic modifications

The importance of these issues, and the risk assessment challenges that each poses, will clearly vary on a case‐by‐case basis. Hence, broad generalizations must be approached cautiously. Nonetheless, the fact that modern agricultural practices deliberately maintain ecosystems in a highly simplified, disturbed and nutrient rich state (Tilman, 1999) suggests that in large, industrial agricultural settings, typical of Canadian and US crop farms (Bokusheva & Kimura, 2016), for example, many of the risk issues under the topic heading ‘nontarget populations’ may be easier to assess than say a release into much smaller agricultural settings, such as Japan, or a natural or semi‐natural environment, since large field size and low crop diversity cause biodiversity to decline in agricultural landscapes (Sirami et al., 2019).

Conversely, there is likely to be heightened concern around the potential for the gene drive‐modified organisms to be toxic or allergenic given the potential for protein residues to enter the food chain, any potential impacts on livestock and other potential risks that are likely very relevant and largely unique to this context, such as those associated with potential changes to land management and agricultural practices that arise due to the availability of the new technology, will additionally need to be considered.

The potential to limit the spread of gene drive‐modified organisms in closely managed agricultural systems should also make the risks easier to assess and potentially lead to mitigation options (Box 2). In our experience, the complexity of an environmental risk assessment increases with its spatio‐temporal scope. If gene drive‐modified organisms can be contained within clearly delineated geographic boundaries, using the genetic and/or physical containment techniques described above and prioritizing high‐threshold gene drive elements, then the risk assessment and the monitoring strategies required to test predictions should be easier to implement. Notwithstanding, a carefully planned postrelease monitoring strategy remains an essential component of any scientific risk assessment if cause‐and‐effect relationships between the release of gene drive‐modified organisms and environmental outcomes are to be discerned with any degree of confidence (NASEM, 2016).

As with the development and ultimate adoption of any new agricultural technology, the risk assessment process cannot focus only on the biophysical uncertainties, but must ultimately incorporate societal acceptance, consumer demand and perceived costs and benefits by communities. The latter can lead to a strong development pipeline for products that can be adopted by farmers or lead to a rejection of new technology before it has even entered the formal risk assessment process. This is especially true for agricultural technologies because the outputs end up in the global food system and because of dual use concerns (Oye et al., 2014) that gene drives against agricultural pests could be used to disrupt food supply. In this review, we have primarily focused on the biological and ecological aspects of gene drive and the associated risks (Table 1); however, any formal evaluation of this new technology will need to account for societal acceptance and understanding of these approaches.

BOX 2 **Gene drive confinement strategies.**

The risk of a gene drive system ‘escaping’ from a target population to a nontarget population is a function of (i) the threshold characteristics of the drive system being implemented, (ii) the migration rate of the species of interest from the target to nontarget populations and (iii) the habitat suitability of nearby nontarget populations. Strategies to mitigate escape include using a drive system with high threshold characteristics and using additional biological and ecological control measures. Potential biological control measures include the following: (i) using promoters of gene drive activity activated by a chemical that is present in the release setting, but not elsewhere, (ii) including a lethality gene in the construct activated by a chemical that could be released in surrounding areas and that other organisms are not affected by and (iii) preventing spread to nontarget species by using genes or promoters that are only functional in the target organism. Potential ecological control measures include the following: (i) choosing a trial site that is not contiguous to another habitat for the target organism (ideally, the nearest habitat would be beyond the normal dispersal distance of the organism) and (ii) implementing other control measures (e.g. traps and insecticides) surrounding the field site. Regarding threshold properties, we may think of each drive system as having a characteristic ‘release threshold’, above which it will spread into a randomly mixing target population, and a ‘migration threshold’, denoting the migration rate at which it will spread into a randomly mixing nontarget population. Marshall and Hay (2012) calculated these thresholds for a range of drive systems and, using a source model and default parameter values (a 5% fitness cost per allele), found the migration thresholds for chromosomal translocations, two‐locus engineered underdominance and single‐locus engineered underdominance to be 4.3%, 2.8% and 17.0% per generation, respectively. All of these migration thresholds likely exceed migration rates between target and nontarget populations in many agricultural settings, although translocations pose a smaller escape risk than two‐locus engineered underdominance, for instance.

## 5 CONCLUSIONS AND PERSPECTIVES

Given the need for innovative and paradigm‐breaking solutions for pest control and resistance management in agricultural systems, it is no wonder that gene drives have been the subject of much recent attention. As we describe above, gene drives have the potential to provide significant benefits in terms of control of undesirable species, flexible management of resistance at the landscape scale and an overall more efficient and targeted use of pesticides. On the other hand, gene drive technologies are highly elaborate and rely on complex interactions between genetic, genomic, biological and ecological specificities of the targeted organism. For all those reasons, and given the wide taxonomic range of potential targets in agricultural environments, significant uncertainty remains regarding the feasibility, suitability and acceptability of these methods, and the path to application for such control solutions is, at the time of publication of this article, obviously very long.

At this stage of product research and development, it is therefore crucial to define and ask these questions, identify the issues and open discussions with all stakeholders, and strive for evidence‐based answers, before committing significant efforts, both human and financial, to projects that may not ever come to fruition. Furthermore, the open sharing of knowledge between scientists and research organizations is even more critical to predicting when and where gene drives will be unsuccessful and likely to waste limited research funds. Understanding cases in which gene drives were not successfully developed is equally as important as identifying those that were successful.

In this review, we have highlighted the specificities of agricultural systems, and how, in several ways, they could offer opportunities for the design and deployment of specific, tailored gene drive approaches. Naturally, many questions remain to be addressed, some regarding our fundamental understanding of the dynamics of gene drive in relation to the ecological and environmental dynamics of the target species, and some related to the plethora of socio‐economic issues associated with gene drives. Ultimately, the fate of any gene drive will depend on a complex set of factors, between its inherent capacity to spread and its ability to be confined if and when needed, between the durability of the engineered construct and the rate of generation of resistant alleles, between traits of the target species and its environment that will facilitate the spread of a drive and traits that will hinder it. Pest populations in agricultural environments interact with natural populations (of different species or of the same species), and consequently, any control method directed at pest populations in managed environments must be evaluated in an area‐wide context, in terms of both success of the control programme and its associated risks. Gene drives will most certainly not provide a silver bullet against all targets in all environments, but might prove a valuable addition to the arsenal of integrated control methods in some specific cases.

With such a multidimensional eco‐evolutionary landscape, quantitative theoretical tools will prove invaluable and will need to account for the biology and ecology of the target species and its environment to be able to evaluate the value of gene drives as a control method. There is a long road of theoretical and experimental research ahead before the value of gene drives in agricultural systems will be realized. Because the areas of uncertainty are manifold, an integrated approach combining experimental investigations of gene drive designs and engineerability, theoretical modelling of population‐ and landscape‐level dynamics of various drives in diverse targets, and socio‐economic analyses of acceptability and regulatory frameworks will be required.

## CONFLICT OF INTEREST

The authors declare that there is no conflict of interest.

## Data Availability

There is no data associated with this article.
